# From Grammont to a New 135° Short-Stem Design: Two-Hand Lever Test and Early Superior–Lateral Dislocations Reveal Critical Role of Liner Stability Ratio and Stem Alignment

**DOI:** 10.3390/jcm14061898

**Published:** 2025-03-11

**Authors:** Stefan Bauer, Jaad Mahlouly, Luca Tolosano, Philipp Moroder, William G. Blakeney, Wei Shao

**Affiliations:** 1 Service d’Orthopédie, Centre de l’épaule et du coude, EHC, 1110 Morges, Switzerland; 2 Medical School, University of Western Australia, Perth, WA 6009, Australia; 3 Department of Shoulder and Elbow Surgery, Schulthess Clinic, 8008 Zurich, Switzerland; 4 Department of Orthopaedics, Royal Perth Hospital, Perth, WA 6000, Australia

**Keywords:** new Perform stem, 135° neck–shaft angle, Grammont, liner stability ratio, reverse shoulder arthroplasty, instability, dislocation, intraoperative testing, Bauer test

## Abstract

**Background**: In reverse shoulder arthroplasty (RSA), the neck–shaft angle (NSA) has trended downward from 155° to 135° to reduce scapular notching, but concerns about instability persist. To assess superior–lateral stability, we developed the intraoperative two-hand lever test (2HLT). The primary objective was to evaluate the effectiveness of the 2HLT, analyze the learning curve in this first study reporting on the new Perform stem, and compare the liner characteristics of 155° and 135° systems. **Methods**: In a single-surgeon learning curve study, 81 RSA procedures with the new Perform stem (Stryker) were included. The outcomes included the 2HLT test applied in 65 cases, early dislocations, stem alignment, stem length, liner type/thickness, and complications. The early dislocation rate was compared to 167 prior Ascend Flex RSA procedures (Stryker). The liner characteristics of three 135° systems (Perform/Stryker, Univers/Arthrex, and Altivate/Enovis) were compared to traditional 155° Grammont systems (Delta Xtend/DePuy, Affinis Metal/Mathys, SMR 150/Lima, and Aequalis Reversed/Stryker), focusing on jump height (JH) and the liner stability ratio (LSR). **Results**: In 63% (31/49) of the cases, the 2HLT detected superior–lateral instability, necessitating a retentive 135° liner. The early dislocation rate in the Perform cohort was 4.9% (0% for retentive liners, 8% for standard liners) versus 0% in the Ascend Flex cohort. The mean effective NSA was 133° (127–144°) for short Perform stems and 135° (129–143°) for long stems. Long Perform stems significantly reduced varus outlier density below 132° and 130° (*p* = 0.006, 0.002). The 36 mm Perform 135° standard liner has a JH of 8.1 mm and an LSR of 152%, markedly lower than the Altivate (10.0 mm/202%) and Univers (9.7 mm/193%) and similar to traditional 155° Grammont liners (8.1–8.9 mm/147–152%). Perform retentive liners have LSR values of 185–219%, comparable to the established 135° design standard liners (195–202%). In the Perform cohort, early complications included four superior–lateral dislocations (all standard liners, LSR 147–152%) requiring four revisions. **Conclusions**: Perform standard liners have a lower LSR than the established 135° designs. Retentive Perform liners (LSR > 184%) are comparable to standard liners of established 135° designs and effectively mitigate instability. We recommend discontinuing non-retentive Perform standard liners (NSA 135°, LSR < 158%) due to the 63% superior–lateral instability rate detected with the novel 2HLT, necessitating retentive liners, the documented LSR-NSA implant mismatch, and an early clinical dislocation rate of up to 8%.

## 1. Introduction

In recent years, reverse shoulder arthroplasty (RSA) has undergone significant advancements in implant design [1]. There has been a transition from the traditional Grammont neck–shaft angle (NSA) of 155° to 145°, now widely adopted in clinical practice. A further reduction to 135° offers potential benefits, including an enhanced range of motion (ROM) and reduced scapular notching, as supported by early clinical reports and computer modeling studies [2,3,4,5,6]. However, these benefits are accompanied by concerns about implant stability, and there is a need for a critical evaluation of a lower NSA [4]. Systematic reviews indicate that the traditional medialized Grammont design has a dislocation rate of 4% compared to 1.3% in non-Grammont implants [7]. Despite these findings, several series of bony-increased-offset (BIO) RSA with NSAs of 155° or 145° report no events of dislocations requiring revision [8,9,10]. Initial studies suggested that lowering the NSA to 135° would not compromise anterior stability [11,12]. However, recent data from a BIO-RSA series utilizing a 135° NSA with a semi-inlay platform stem and eccentric glenosphere revealed a dislocation rate of 3.8%, raising concerns about the stability associated with a low NSA [4]. These findings highlight the need to explore the mechanisms of instability associated with a lower NSA. Dislocation patterns may have shifted with the adoption of 135° NSA designs. While anterior and anterior–superior instability were predominant with a higher NSA, superior–lateral dislocations may now be more prevalent, particularly in cases of varus alignment.

Frankle proposed a classification system for RSA instability requiring revision, identifying factors such as loss of compression, containment, and impingement [13]. However, this framework does not address the influence of a lower NSA or varus alignment on dislocation direction, leaving a critical gap in understanding this etiology. Moroder et al. were the first to show the large variability in liner constraint across implant systems [14]; however, to date, liner constraint has not been classified according to implant design groups, and it may adversely affect RSA stability, according to Frankle’s classification, through insufficient containment in superior–lateral direction [13].

**Figure 1:**
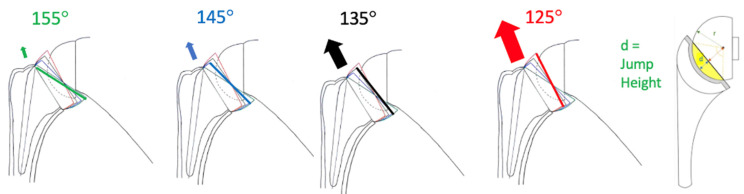
The thickness of the arrows indicates susceptibility to superior–lateral instability with an increasing effective NSA. Illustration of jump height (d) and radius of the glenosphere (r) on the right.

The introduction of the Stryker Perform humeral short stem, featuring a 135° NSA and reduced distal canal filling ratio, has highlighted these concerns as its susceptibility to varus alignment may be linked to superior–lateral dislocations (Figure 1).

**Figure 2:**
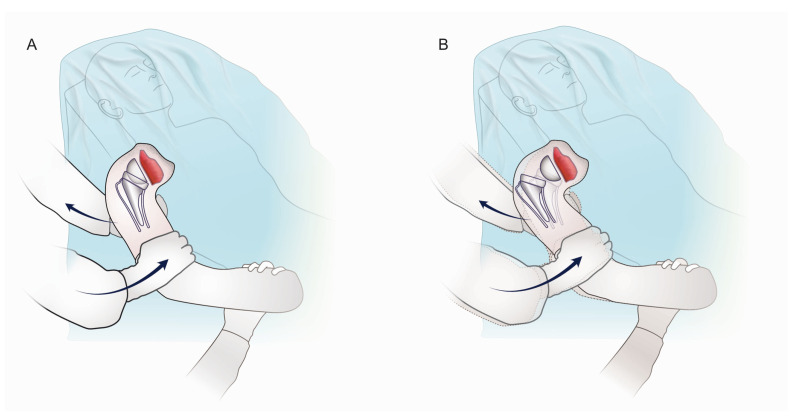
The 2HLT/Bauer test is a sensitive test for detecting superior–lateral instability associated with a more vertical joint line in 135° RSA. (**A**) Stable, negative test. (**B**) Unstable, positive test with dislocation.

These challenges emphasize the importance of intraoperative testing to comprehensively assess RSA stability. Currently, intraoperative methods for evaluating joint reaction forces and detecting instability are limited [1]. Tests such as external rotation in the neutral position, abduction/external rotation, and adduction/internal rotation are used to address anterior, posterior, and anterior–superior instability. However, they do not specifically target superior–lateral stability. Other described tests include the “shuck test” (pistoning), “bed shuffle test” (antero-superior instability), and “lateral thrust test” (lateral instability via a non-physiological intra-articular thrust test) [15]. To address this gap, we propose the two-hand lever test (2HLT), a novel intraoperative method designed to detect superior–lateral instability in RSA with a 135° NSA. This test evaluates stability by simulating superior–lateral forces and by challenging the liner’s jump height through an indirect lever mechanism mimicking physiological upper arm impingement, such as during the compression of the arm against the thorax in some flexion (Figure 2). The 2HLT provides a practical tool for surgeons to identify instability and optimize liner thickness and constraint selection intraoperatively.

This study aims to evaluate the effectiveness of the 2HLT in identifying superior–lateral instability, thus guiding liner type and thickness selection. The secondary objectives include analyzing early radiographic stem alignment to assess the learning curve for the new 135° short stem and comparing liner constraint characteristics between common 155° and 135° systems.

## 2. Materials and Methods

In this learning curve study on consecutive patients, a total of 81 RSA procedures in 55 females and 26 males (mean age: 74, 54–89; demographics are shown in Table 1) were carried out by the senior author (SB), a shoulder arthroplasty surgeon with 10 years of arthroplasty experience. The new Perform humeral stem (Stryker, Kalamazoo, MI, USA) was combined with either a lateralized Perform baseplate (Stryker, Kalamazoo, MI, USA) as a MIO-RSA (metal-increased offset) or with a Reversed II baseplate (Stryker, Kalamazoo, MI, USA) as a BIO-RSA (bony-increased offset) procedure with a baseplate lateralization of 5–12 mm. As a comparative series, 167 consecutive preceding RSAs in 118 females and 49 males (mean age: 75, 57–91; Table 1), treated with an Ascend Flex stem (Stryker, Kalamazoo, MI, USA) combined with either a lateralized Perform baseplate as a MIO-RSA or with a Reversed II baseplate as a BIO-RSA, were analyzed for an effective NSA and early instability within 90 days after surgery. These operations were all carried out by the same surgeon (SB). All cases were planned with 3-D planning software (Blueprint 4.0.1, Imascap, Brest, France), targeting an anatomic humeral lateralization of +0 to +2 mm, as recommended in previous studies [16]. For the Perform stem, an intramedullary alignment rod-guide assembly was used in all cases, aiming for a 135° humeral osteotomy in 20–30° of retrotorsion. The Ascend Flex short stem was implanted using a semi-inlay technique with a 145° NSA (stem B, liner B) without repair of the subscapularis tendon in 136 of 167 cases (81%). A subscapularis repair was performed in 31 cases (19%). No cases from the Perform or Ascend Flex series were excluded or influenced by specific inclusion or exclusion criteria, as no such criteria were applied.

### 2.1. Two-Hand Lever Test

**Figure 3:**
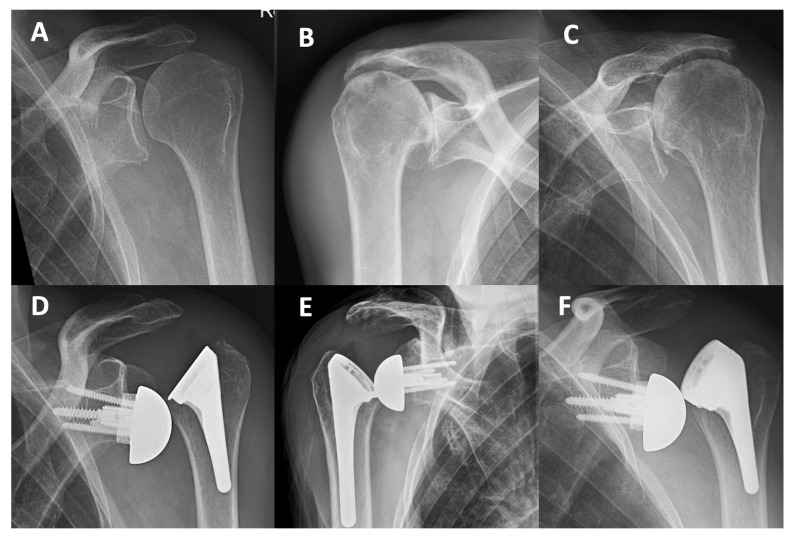
Preoperative radiographs of three patients who sustained dislocations (**A**–**C**). Superior–lateral dislocations of short Perform stems in combination with standard non-retentive liners (**D**–**F**).

As an adverse event, the 9th Perform case dislocated 5 weeks after surgery, which was initially managed with a closed reduction. However, a second dislocation within three days required open revision surgery. The implants used were a short-size 2+ stem with a recommended osteotomy height of +0 to +4 mm above the anatomical neck of the humerus, a symmetric insert of +3 mm height, as generally recommended as an optimized insert for the Perform stem to achieve balance, and a 39 + 3 mm eccentric glenosphere on a 25 + 2 (inbuilt compared to Reversed II baseplate) + 6 mm lateralized base plate, as shown in Figure 3D. Postoperative stem alignment of 6° varus was measured, as shown in Figure 3D, F. During revision surgery, the subscapularis tendon repair was found to be intact, and there was no anterior instability in external rotation (ER). After reducing the humeral component of the RSA, the arm was removed from the arm holder and held by the assistant in 30° of flexion. Superior–lateral dislocation of the prosthesis was detected by placing four fingers of the surgeon’s flat hand medial to the proximal humerus, near the axilla, while applying a superior–lateral force. Simultaneously, the second hand applied a medially directed force on the distal humerus (see Figure 2 and Supplementary Video S1). We named this 2-handed, intraoperative test without intra-articular manipulation the two-hand lever test/Bauer test (2HLT, Figure 2), described as a novel test to detect superior–lateral instability or the “disengagement” of an RSA with a 135° NSA or even a lower effective NSA in case of unnoticed varus alignment of the stem leading to a verticalized joint line (Figure 1; 125° in red). The 2HLT simulates an indirect lever mechanism that patients may encounter during adduction of the flexed arm against the thorax. This intraoperative test was performed by both the surgeon (SB) and the shoulder fellow in all subsequent Perform stem cases. Both observers performed the test twice. If the RSA did not dislocate laterally after 2 testing maneuvers by both examiners, the 2HLT was recorded to be negative. It was recorded as positive after the occurrence of a superior–lateral dislocation with a +0 mm standard liner. The test was then repeated with a +3 mm thicker liner. Testing by the observer was discontinued once the 2HLT was negative with a standard or retentive liner. Under complete muscle relaxation, the first liner tested was always a +0 mm standard liner prior to testing with a +3 mm standard liner.

A consecutive series of 65 Perform stem RSAs was examined by the senior surgeon (SB) and shoulder fellow using the 2HLT. The consensus results of the 2HLT were recorded, as well as the final implant choice in terms of insert thickness (+0, +3, +6, +/− additional spacer), liner angle (symmetric or +10° angle), and whether a retentive or standard liner was used.

### 2.2. Stem Alignment and Relationship to Stem Length

Standard true ap radiographs of the proximal humerus were obtained postoperatively during admission, at 6 weeks, and at 3 months. The stem alignment was measured by the surgeon (SB) and the fellow. The stem alignment and effective NSA were recorded as follows. If the measurements differed by less than 3°, their mean was recorded. For discrepancies greater than 3°, the senior investigator (SB) performed a second measurement, and the median of three measurements was recorded to eliminate potential outliers.

The density distribution of effective neck–shaft angles (NSAs) was analyzed for both the short and long Perform stems.

### 2.3. Review of Liner Stability

Having identified a new direction of superior–lateral instability with the 2HLT in association with the 135° Perform short stem design (Stryker, Kalamazoo, MI, USA), we aimed to review the RSA liner stability ratios across a variety of implant systems. These included traditional Grammont RSA systems such as the Delta-Xtend (Depuy, Warsaw, IN, USA), Aequalis Reversed II (Stryker/Tornier, Montbonnot, France), Affinis Metal 147° (Mathys, Bettlach, Switzerland), and SMR (Sistema Modulare Randelli) 150° (Lima, San Daniele, Italy). Additionally, we analyzed established 135° systems that have been in clinical use for more than five years, including the Altivate (Enovis, Austin, TX, USA) and Univers system (Arthrex, Naples, FL, USA).

The liners of the new Perform stem design were also compared alongside three generations of Stryker/Tornier RSA implants spanning over 30 years of development: the 155° Aequalis Reversed design from the 1990s, the Aequalis Ascend Flex 145° design launched in 2014, and the Perform 135° design introduced in 2021.

The liner jump height (JH), which is identical to liner depth (d), as illustrated in Figure 1 and Table 2 and Table 3, was used to calculate the liner stability ratio (LSR) and angle of coverage (AOC) using the formulae that were first published and applied to RSA by Moroder et al. [LSR = (square root (1 − (r − d/r)^2^)) / (r − d/r); Angle of Coverage = 2arccos(1 − d/r) × 180/π] [14].

The Altivate system, introduced by Frankle in the early 2000s, served as the most established reference for the 135° design. This implant is predominantly used with 32 mm and 36 mm glenosphere sizes. To ensure a meaningful comparison, we graphically analyzed similar-sized Stryker/Tornier implant generations and 135° designs, as well as traditional Grammont 155° systems.

### 2.4. Statistics

Descriptive statistics were used to analyze the clinical and radiographic data. A chi-square test of independence was performed to compare the prevalence of varus outliers (effective NSA < 132° and <130°) between the long and short Perform stems.

This test was chosen because it is well suited for categorical data, allowing us to assess whether the observed distribution of varus outliers deviated from expected proportions. While any degree of varus misalignment is a concern, we arbitrarily selected deviations greater than 3° as clinically concerning and deviations greater than 5° as a serious issue. These thresholds ensured a structured comparison of stem performance based on meaningful biomechanical deviations.

The chi-square test was deemed appropriate as it evaluates the independence between categorical variables and is suitable for comparing frequency distributions across independent groups. Statistical significance was set at *p* < 0.05.

## 3. Results

**Figure 4:**
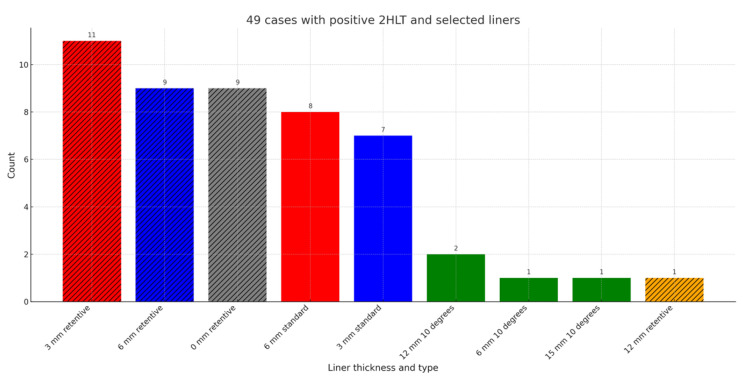
Selected liners after positive 2HLT testing.

Of 65 RSA cases with the new Perform stem combined with glenoid lateralization after introduction of the 2HLT, 49 cases (75%) were positive, defined as a positive test with a +0 mm standard liner. The 2HLT influenced implant selection in these cases. Testing with the 2HLT commenced after the detection of superior–lateral instability in the ninth case of the series, following the implantation of 16 cases. Figure 4 illustrates the liner types used after a positive 2HLT. The most frequently used liners were retentive liners (30 out of 49 cases, 61%).

Prior to the commencement of 2HLT testing, a retentive liner was used in 1/16 cases (6%), which increased to 30/65 cases (46%). A long stem was used in the first cohort in 2/16 cases (13%), which increased to 47/65 cases (72%) in consecutive patients.

Of the 35 short Perform stems, 10 (29%) had varus outliers below 130°, and an additional 5 (14%) had varus outliers below 132°, making a total of 15 (43%) below 132°.

**Figure 5:**
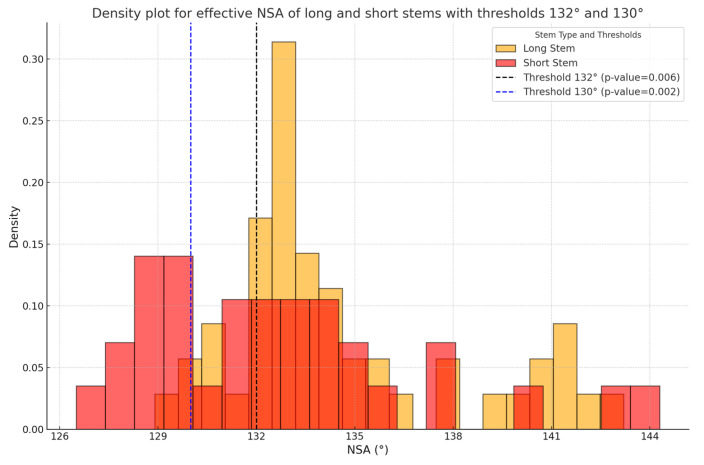
Density plot for the effective NSA of the long and short Perform stems with thresholds below 132° and 130°, showing a significantly higher number of varus outliers with the short stems.

Of the 49 long Perform stems, 2 (4%) had varus outliers below 130 degrees, and an additional 5 (10%) had varus outliers below 132 degrees, making a total of 7 (14%) below 132 degrees. The mean effective NSA was 133° (127–144°) for the short stems and 135° (129–143°) for the long stems. The long stems showed a significantly reduced varus outlier density below the thresholds of 132° (14%, 7/47 vs. 43%, 15/35) and 130° (4%, 2/47 vs. 29%, 10/35), with *p* = 0.006 and *p* = 0.002, respectively, as shown in Figure 5. The Ascend Flex short stems had a mean effective NSA of 154° (139–160°), with 92% of the stems showing valgus alignment.

### 3.1. Early Superior–Lateral Instability and Complications

Major early complications in the Perform cohort (minimum follow-up of 90 days) included four superior–lateral dislocations in three patients (Table 4).

The indication for RSA was CTA in two of these patients, and one had a massive cuff tear (Figure 3). In one of the four cases, the subscapularis had been repaired, was still intact at revision, and did not prevent superior–lateral instability. This first dislocation (case 1, Table 4) was revised to a +9 mm spacer with a +3 mm standard 10° 39 mm insert (LSR 147%) and dislocated again, requiring a second revision (case 2, Table 4). A varus alignment of 6° (effective NSA 129°) was considered to contribute to the superior–lateral instability (Figure 3D,F) and corrected (stem revision of case 2) and combined with +6 mm 10° standard liner +9 mm spacer (+15 mm in total). The glenosphere was changed from a 39 mm eccentric to a centered 42 mm implant. In summary, superior–lateral instability occurred in 8% of the cases with a standard non-retentive liner (4/50), LSR < 158%). Three liner-change revisions and a major revision involving both a liner, glenosphere and stem exchange to correct the varus alignment (129°) were necessary to achieve stability. The contributing factors affecting instability in the four adverse dislocation events were low LSR (four times), liner type (three times), MRCT or CTA (four times), varus alignment (one time), and low compression (one time).

Additionally, two cases of temporary axillary nerve paresis (3.1%) were reported. Notably, no cases of early instability were observed in the Perform cohort when a retentive liner (LSR > 184%) was implanted, nor in the Ascend Flex cohort with a mean effective NSA of 154°. In the Ascend Flex cohort, four patients sustained a scapular spine fracture, and three were treated with open reduction and internal fixation, as previously published [17].

### 3.2. Review of 155° Grammont and 135° Design Liners

The liner characteristics of three 135° systems—Perform (Stryker), Univers (Arthrex), and Altivate (Enovis)—were compared with those of traditional 155° Grammont systems, including Delta Xtend (DePuy), Aequalis Reversed (Stryker/Wright/Tornier), Affinis Metal 147 (Mathys), and SMR 150 (Lima). The analysis focused on two key biomechanical parameters: jump height (JH), a measure of resistance to dislocation, and the liner stability ratio (LSR), which quantifies the mechanical retention capabilities of the liner [14].

**Figure 6:**
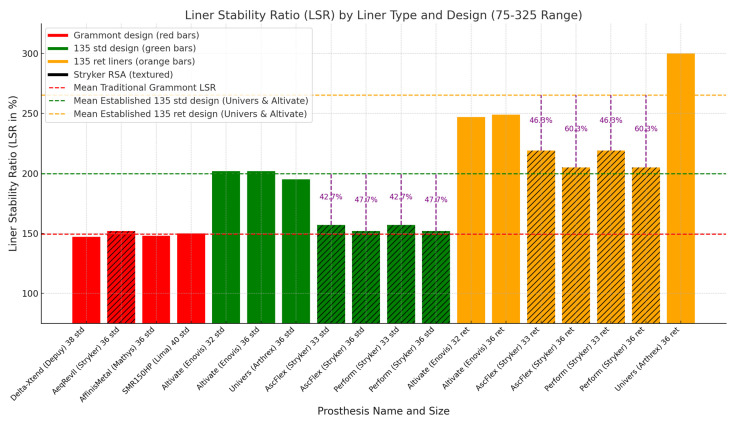
Liner stability ratio (LSR) for Grammont 155° std liners (red), 135° std liners (green), 135° ret liners (orange), and Stryker RSA (textured). Dashed mean trendlines for mean traditional Grammont design (red), established 135° standard (green), and ret design (orange), std = standard, ret = retentive, liner size in mm.

Figure 6, along with Table 2 and Table 3, illustrates the differences in the JH and LSR across these systems. Standard liners of the established 135° designs demonstrated superior JH and increased LSR compared to both the new Perform 135° design standard liners and the 155° Grammont systems. Notably, the LSR of the new Perform 135° design standard liners (36 mm glenosphere) matched that of the 145° Ascend Flex and the 155° Aequalis Reversed Grammont systems (all Stryker/Wright/Tornier), indicating that no significant changes were made in the liner characteristics across three generations of implants, transitioning from the 155° Grammont design to a lateralizing short-stem 145° platform design and, subsequently, to the 135° Perform inlay design.

When comparing the new Perform 135° design standard liners with the standard liners of the established 135° designs (Altivate and Univers), a notable stability gap was observed. The mean JH gap was 1.7 mm (range: 1.47–1.92 mm), while the mean LSR gap was 46% (range: 43–48%). The mismatch in the Perform standard liner LSR with the 135° Perform design is illustrated in Figure 6. These findings underscore measurable differences in the biomechanical properties of the new 135° Perform design relative to the established 135° systems. The Perform retentive liners, however, demonstrated LSR values ranging from 185% to 219%, comparable to those of the established 135° design standard liners (195–202%), effectively mitigating the observed JH and LSR gaps and mismatch in the Perform standard liners.

## 4. Discussion

The key findings of this learning curve study, combined with an in-depth review and new classification of RSA liners, indicate that the clinically investigated new 135° design is prone to a previously undescribed type of RSA instability termed “superior–lateral instability”. This phenomenon was identified and assessed using a novel intraoperative test, the two-hand lever test (2HLT). The 2HLT proved to be a valuable intraoperative tool, yielding positive results in 75% of the consecutive cases where it was applied. Its implementation followed the first observed occurrence of superior–lateral dislocation and significantly influenced implant selection. Specifically, retentive liners (LSR 185–219%) were utilized in 61% (31/49) of cases with a positive 2HLT result. Early superior–lateral dislocations requiring revision surgery were observed in 8% of cases in the Perform cohort with standard liners (LSR < 158%), whereas no early instability events were reported in the 167 Ascend Flex control cohort. Notably, no instances of instability occurred when Perform retentive liners (LSR 185–219%) were employed.

Perform standard liners, with an LSR < 158%, exhibit a measurable gap in both jump height (JH) and LSR compared to established 135° designs that have been in clinical use for over 10 years, such as the Altivate and Univers systems. The standard liners of the 135° Perform design demonstrated an equivalent LSR range (144–157%) to traditional 155° Grammont systems (133–175%), as illustrated in Figure 6. This is the first study to classify and differentiate the LSR and JH by design groups focusing on the NSA (155° Grammont design vs. established 135° designs). The findings highlight the critical role of the LSR and JH in mitigating superior–lateral instability in 135° designs. Furthermore, they underscore the protective effect of retentive liners (LSR of 185–219%) in the 135° Perform design, achieving biomechanical equivalence with established 135° design systems (LSR 195–202%). Grammont designs are inherently more stable in the superior–lateral direction (Figure 1) because the 155° NSA positions the liner inferior–medially under the glenosphere. Therefore, Grammont design liners do not require the same JH and LSR as 135° liners. In this study, the LSR gap and mismatch in the Perform 135° standard liners resulted in an increased short-term dislocation and revision rate, which may influence the long-term outcomes of affected patients. Over time, additional dislocations and revisions may occur, further impacting the long-term outcomes. Therefore, meticulous long-term follow-up is necessary.

The wide variation in LSRs across manufacturers has only recently been highlighted by Moroder et al. [14]. However, its clinical implications have not been reported in the literature. To our knowledge, this is the first study to relate LSRs to the NSA of implants, establishing a clinical context for instability, dislocations, and increased revision rates.

Varus alignment outliers were not infrequently associated with the short Perform stems (29% < 130° and 14% < 132°) even after intramedullary-guided osteotomies. One patient required a stem revision for varus malalignment with instability.

There is a paucity of literature describing intraoperative stability testing and the evaluation of joint reaction forces during RSA trialing. Javed et al. proposed multiple intraoperative tests, including external rotation in a neutral arm position (hinged opening), abduction and external rotation (anterior dislocation), adduction and internal rotation (posterior dislocation), conjoint tendon tension assessment (evaluating excessive or insufficient tension), the “shuck test” (excessive pistoning), the “bed shuffle test” (anterior–superior dislocation), and the “lateral thrust test” (lateral dislocation) [15]. The lateral thrust test applies a laterally directed force with a finger or hook directly on the humeral calcar. While it may be sensitive for detecting lateral dislocations, its intra-articular force application with a hook or finger on the humeral calcar may limit its clinical relevance. In contrast, the two-hand lever test (2HLT) applies a lateral force to the 30° flexed proximal humerus, simulating physiological conditions during the forced adduction of the upper arm against the thorax. To our knowledge, this test is novel in detecting superior–lateral instability and has not been previously described in the literature. The 2HLT directly challenges RSA liner JH and compressive joint reaction forces, both of which are crucial biomechanical variables for RSA stability [18]. In our cohort, 75% of the cases (49/65) had a positive 2HLT, and 63% (31/49) were subsequently treated with a retentive liner (LSR 185–219%).

Previous studies have investigated compressive forces in different arm positions with trial sensors integrated in RSA trial liners, but, to date, no load values in different arm positions have been defined for different RSA designs, and trial insert sensors are currently not available for clinical practice [19].

Instability is among the most commonly reported complications following RSA [20,21,22], with a pooled dislocation rate of 4% [22]. Early RSA dislocations within 90 days of implantation are uncommon, occurring in 2.9% of RSAs without baseplate lateralization [23]. However, in the presented cohort, the early dislocation rate of 8% in cases with standard liners (LSR < 158%) and the prevalence of superior–lateral instability in 75% of cases (positive 2HLT) with a standard liner are concerning.

Patient factors associated with an increased RSA dislocation risk are male gender, BMI > 30 kg/m^2^, subscapularis deficiency, soft tissue pathologies (e.g., Ehler–Danlos), Parkinson’s disease, severe proximal humerus fractures, and previous surgery [20,23,24,25,26,27,28]. Apart from male gender and subscapularis deficiency in 75% of the dislocations in the presented cohort, no other factors were recorded for these adverse dislocation events.

Biomechanical factors were analyzed in a benchmark study, including compressive glenohumeral joint reaction force, jump height (socket depth), and glenosphere size. The study examined the hierarchy of mechanical stability factors, which was led by compressive forces followed by the jump height of the liner. Glenosphere size played a much lesser role [18].

There are several series of bony-increased-offset RSA with an NSA > 135° using the Aequalis Reversed and Ascend Flex design (both Stryker) without any reported dislocations [8,9,10]. This can be explained by the increased baseplate offset [8,9,10], as well as lateralizing curved onlay stem design, both increasing the compressive joint reaction forces [10]. These results are in keeping with the results of our increased offset Ascend Flex cohort, which did not show any early dislocations within 90 days. The Ascend Flex implant with increased baseplate offset has been known to be associated with increased compressive joint reaction forces, often requiring reduction with a “shoehorn” prosthesis reducer in clinical practice, which has been associated with a higher rate of scapular spine fractures. The design combination was classified by Werthel et al. as one of the most lateralizing implant configurations [16]. Lowering the NSA to 135° with the same Ascend Flex configuration has been reported with a dislocation rate of 3.8%, which raises concerns about instability associated with a 135° NSA [4].

Investigating the Perform design evolution by evaluating previous implant generations of the same company (Stryker, Wright, and Tornier), we found that RSA liner characteristics have been kept almost identical over more than 20 years, from Grammont to a new 135° short-stem design. The Aequalis Reversed shoulder prosthesis received FDA (Food and drug administration, Washington, DC, USA) clearance in 2005 and is one of the first classic 155° Grammont design prostheses alongside the Delta-Xtend (Depuy), with a medialized center of rotation (COR). The COR was subsequently lateralized as a bony-increased-offset RSA [8]. The Ascend Flex was approved by the FDA in 2014 and is characterized as one of the most lateralized RSA implant configurations when combined with a metal- or bony-increased-offset of the baseplate [16]. The Perform humeral stem was launched in the USA in 2021 and in Europe in 2022. Taking the Ascend Flex design with combined lateralization into account, the implant design of the Perform has undergone a metamorphosis. The curved onlay Ascend Flex short stem was mainly a 145° design with the option to decrease the NSA to 135°. Undersizing this stem to prevent stress shielding has been shown to be associated with valgus alignment [29]. Our data confirm a tendency toward valgus alignment in the Ascend Flex. Introducing the Perform stem, the design was transformed to a 135° short-stem inlay design with the option to increase the NSA to 145° with a 10° liner. The new Perform inlay short stem was designed to reduce proximal stress shielding by decreasing its distal filling ratio and improving proximal metahyseal loading. However, a reduced distal filling ratio of the stem increases the risk of varus malalignment. Additionally, it was developed to reduce humeral lengthening and distalization, thereby decreasing tension. At the same time, a new 135° design was introduced to enhance impingement-free range of motion (ROM).

The standard liner JH and LSR are either identical (36 mm) or decreased (42 mm) compared to the early 155° Aequalis Reversed Grammont design, identical to the Aequalis Ascend Flex 145° design (Table 2 and Table 3; Figure 6), and not adapted to match the LSR of the established 135° designs, which are reported to have 5-year dislocation rates of 0% and 4% [30,31]. Our study indicates a tendency toward varus alignment in the short Perform stem.

In summary, the new Perform short stem design was designed to decrease distalization and stem-related lateralization, reducing compression across the joint without adapting the JH or LSR, which were kept at the level of the traditional Grammont designs. The biomechanical implant variables, compression, and JH are crucial for RSA stability [18]. As an additional factor, Perform 135° short stems are susceptible to varus outliers below 132° and 130°, as shown in our study, in contrast to the Ascend Flex stem. Varus outliers may verticalize the joint line, further increasing the risk of superior–lateral instability (Figure 1). The new Perform stem has not been monitored in the Australian or Scandinavian joint registries as yet. All the dislocations had a positive 2HLT. This was addressed intraoperatively by using a thicker liner to increase stability, but not a retentive liner. Based on the early dislocation rate, the intraoperative findings of superior–lateral instability, and the liner stability ratio gap presented in this study, we have reported standard Perform liners to the national regulatory authority of therapeutic products (Swissmedic report 5102, 05/02/2025) with the recommendation to discontinue the use of the standard liners of the investigated implant system with an LSR of 144–157% (Table 2) and not to use them at all. With the implant investigated in this study, the use of a retentive liner (LSR 185–219%) was not associated with any dislocations in our series. It was shown to provide equivalent impingement-free motion compared to standard 10° valgus liners [32], as long as implant positioning and glenosphere overhang are optimized [33].

This study has several strengths. It reports early clinical and intraoperative findings using a novel test for a new 135° implant, offering valuable insights into design-related instability. Furthermore, the classification of the LSR and JH by NSA design groups provides a new framework for evaluating RSA liners.

However, there are limitations. First, the single-surgeon study design ensures procedural consistency but limits generalizability. Second, we were unable to blind the 2HLT for reliability testing in the intraoperative setting. However, without available objective systems to measure compressive forces across the joint, the 2HLT is a useful test to assess the susceptibility for superior–lateral instability challenging the JH, LSR, and compressive forces in superior–lateral direction. Combining this clinical test with measurements of compressive forces (sensors in liners) may shed light on the required combination of compressive forces and liner constraints in the future. Finally, the minimum follow-up was short and restricted to 90 days. The exact same follow-up period was previously investigated in a study that serves as a reference for early RSA instability [23]. However, we acknowledge that this limitation prevents an estimation of mid-term and long-term dislocation rates, particularly when this implant is used with standard liners. Additionally, assessing the efficacy of retentive liners requires a longer follow-up. Therefore, we emphasize the need for extended studies to further evaluate implant stability and complication rates over time.

## 5. Conclusions

This is the first study to classify liner JH and LSR based on NSA design groups (155° Grammont vs. established 135° designs). We recommend discontinuing non-retentive Perform standard liners (NSA 135°, LSR < 158%; Swissmedic reference 5102, 05/02/2025) due to the previously under-reported phenomenon of superior–lateral instability. This implant-related instability, effectively detected by the novel 2HLT, necessitated the use of retentive liners (LSR > 184%) in 63% of the cases with a 135° NSA. This highlights the documented LSR-NSA mismatch and stability gap, which contribute to a clinical dislocation rate of up to 8%.

## Figures and Tables

**Table 1:** Patient demographics of the Perform and Ascend Flex cohorts. Cuff Tear Arthropathy (CTA), Massive Rotator Cuff Tear (MRCT), Osteoarthritis (OA), reverse shoulder arthroplasty (RSA).

	81 Perform Stem RSA	167 Ascend Flex Stem RSA
Mean age in years (range)	74 (54–89)	75 (57–91)
Gender		
Female	55 (68%)	118 (71%)
Male	26 (32%)	49 (29%)
Diagnosis		
CTA	35 (43%)	69 (41%)
MRCT	22 (27%)	56 (34%)
OA	24 (30%)	31 (19%)
Others	0	11 (7%)

**Table 2:** Standard and retentive liner characteristics for 135° implant designs, std = standard, ret = retentive, * exact liner depth communicated by the companies.

Liner Type	Jump Height d (mm)	Liner Stability Ratio (%)	Angle of Coverage (°)
Altivate (Enovis) 32 std *	8.90	202	127
Altivate (Enovis) 32 ret *	10.00	247	138
Altivate (Enovis) 36 std *	10.00	202	127
Altivate (Enovis) 36 ret *	11.30	249	136
Aequalis Ascend Flex (Stryker) 33 std *	7.65	157	115
Aequalis Ascend Flex (Stryker) 33 ret *	9.65	219	131
Aequalis Ascend Flex (Stryker) 36 std *	8.10	152	113
Aequalis Ascend Flex (Stryker) 39 ret *	10.10	205	128
Aequalis Ascend Flex (Stryker) 39 std *	8.55	147	112
Aequalis Ascend Flex (Stryker) 39 ret *	10.55	194	125
Aequalis Ascend Flex (Stryker) 42 std *	9.00	144	110
Aequalis Ascend Flex (Stryker) 42 ret *	11.00	185	123
Perform (Stryker 33 std *	7.65	157	115
Perform (Stryker) 33 ret *	9.65	219	131
Perform (Stryker) 36 std *	8.10	152	113
Perform (Stryker) 36 ret *	10.10	205	128
Perform (Stryker) 39 std *	8.55	147	112
Perform (Stryker) 39 ret *	10.55	194	125
Perform (Stryker) 42 std *	9.00	144	110
Perform (Stryker) 42 ret *	11.00	185	123
Univers (Arthrex) 36 std *	9.80	195	126
Univers (Arthrex) 36 ret *	12.30	300	143
Univers (Arthrex) 42 std *	11.40	195	126
Univers (Arthrex) 42 ret *	13.90	278	140

**Table 3:** Standard liners for established Grammont 155° implants and related designs, std = standard, * exact liner depth communicated by the companies, ** measured liner depth from planning software [14].

Liner Type	Jump Height (mm)	Liner Stability Ratio (%)	Angle of Coverage (°)
Delta-Xtend (Depuy) 38 std **	8.30	147	112
Delta-Xtend (Depuy) 42 std **	9.40	151	123
Aequalis Reversed II (Stryker) 36 std *	8.10	152	113
Aequalis Reversed II (Stryker) 42 std *	10.60	175	121
Affinis Metal 147 (Mathys) 36 std **	7.90	148	112
Affinis Metal 147 (Mathys) 42 std **	10.4	171	119
SMR 150 (Lima) 40 std **	8.90	150	113
SMR 150 (Lima) 44 std **	8.80	133	106

**Table 4:** Patient characteristics, implant configuration, and stem alignment of the four dislocations requiring four revision procedures. G (Gender), Diag (Diagnosis), BP (baseplate), glenosphere (GS), eccentricity (ecc).

Age	G	Diag	BP Size and Offset	GS Size and ecc	Stem Size and Length	Liner Type and Thickness Before Dislocation	LSR	Effective NSA	Frankle Classification [13] 1. Compression 2. Containment 3. Impingement	2HLT	Revised to
79	M	MRCT	25 mm +8 mm	39 mm +3 mm	2+ short	Standard +3 mm	147%	129°	1. Low 2. LSR: 147% 3. None	+	Standard 10° +12 mm
79	M	MRCT	25 mm +8 mm	39 mm +3 mm	2+ short	Standard 10° +12 mm	147%	139°	1. High 2. LSR: 147% 3. None	+	Standard 10° Effective NSA 145°, 42 mm +15 mm
84	M	CTA	25 mm +10 mm	36 mm +2 mm	3+ long	Standard +6 mm	152%	134°	1. Normal 2. LSR: 152% 3. None	+	Standard +12 mm
77	F	CTA	25 mm +10 mm	36 mm +2 mm	2+ long	Standard +6 mm	152%	134°	1. Normal 2. LSR: 152% 3. None	+	Retentive +12 mm

## Data Availability

Direct access to the data of this study is not available due to privacy and ethical restrictions.

## Supplementary Materials

The following supporting information can be downloaded at https://www.mdpi.com/article/10.3390/jcm14061898/s1: Video S1: 2HLT.
